# The Impact of Community Shuttle Services on Traffic and Traffic-Related Air Pollution

**DOI:** 10.3390/ijerph192215128

**Published:** 2022-11-16

**Authors:** Zilong Zhao, Mengyuan Fang, Luliang Tang, Xue Yang, Zihan Kan, Qingquan Li

**Affiliations:** 1 State Key Laboratory for Information Engineering in Surveying, Mapping and Remote Sensing, Wuhan University, Wuhan 430079, China; 2 School of Geography and Information Engineering, China University of Geosciences, Wuhan 430074, China; 3 Department of Geography and Resource Management, The Chinese University of Hong Kong, Shatin, Hong Kong, China; 4 Institute of Space and Earth Information Science, The Chinese University of Hong Kong, Shatin, Hong Kong, China; 5 College of Civil and Transportation Engineering, Shenzhen University, Shenzhen 518060, China

**Keywords:** community shuttle services, traffic congestion alleviation, traffic emissions, origin–destination data, eco-friendly transportation, big data

## Abstract

Community shuttle services have the potential to alleviate traffic congestion and reduce traffic pollution caused by massive short-distance taxi-hailing trips. However, few studies have evaluated and quantified the impact of community shuttle services on urban traffic and traffic-related air pollution. In this paper, we propose a complete framework to quantitatively assess the positive impacts of community shuttle services, including route design, traffic congestion alleviation, and air pollution reduction. During the design of community shuttle services, we developed a novel method to adaptively generate shuttle stops with maximum service capacity based on residents’ origin–destination (OD) data, and designed shuttle routes with minimum mileage by genetic algorithm. For traffic congestion alleviation, we identified trips that can be shifted to shuttle services and their potential changes in traffic flow. The decrease in traffic flow can alleviate traffic congestion and indirectly reduce unnecessary pollutant emissions. In terms of environmental protection, we utilized the COPERT III model and the spatial kernel density estimation method to finely analyze the reduction in traffic emissions by eco-friendly transportation modes to support detailed policymaking regarding transportation environmental issues. Taking Chengdu, China as the study area, the results indicate that: (1) the adaptively generated shuttle stops are more responsive to the travel demands of crowds compared with the existing bus stops; (2) shuttle services can replace 30.36% of private trips and provide convenience for 50.2% of commuters; (3) such eco-friendly transportation can reduce traffic emissions by 28.01% overall, and approximately 42% within residential areas.

## 1. Introduction

With accelerated urbanization, the newly built-up areas of cities are rapidly expanding, far beyond the coverage of public transport services [1]. As a result, residents living on the outskirts of the city have to access the nearest public transport hub by self-driving and taxi-hailing (e.g., taxi, Uber, and DiDi) [2]. These massive personal short-distance trips impose certain travel costs on travelers, cause considerable stress on road traffic, and further deteriorate the surrounding environment [3].

According to the China Mobile Source Environmental Management Annual Report [4], mobile source pollution has become an important source of air pollution in large- and medium-sized cities in China, causing serious environmental degradation and health hazards. Among them, automobiles are the main contributors to total motor vehicle pollutant emissions, emitting more than 90% of carbonic oxide (CO), hydrocarbon (HC), oxynitride (NOx), and particulate matter (PM). How to mitigate traffic emissions without compromising convenient mobility options and economic growth has become a realistic and urgent issue for urban planners, enterprises, and the public [5].

Community shuttle services, as flexible, customized, emerging transportation means, have the potential to improve the last leg connectivity of the transportation system and fill the gap of sparse public transport services, especially subways [2,6]. Constructing shuttle services and encouraging residents to shift travel patterns from private modes to shuttle services may alleviate traffic pressure and further provide cost-effective and environmentally friendly transportation for citizens [7].

So far, studies related to community shuttle services are mainly focused on methodologies, especially on route network design. Since the network design problem is an NP-hard problem, it is difficult to solve the problem directly and efficiently. Thus, some heuristic algorithms have been adopted to obtain satisfying results. Kuan et al. [8], Xie, Gong and Wang [6], and Mohammed et al. [9] proposed route network design methods based on the genetic algorithm and ant colony optimization algorithm to minimize the route length or maximize service capacity. Tong et al. [10], Kong et al. [11], Tong et al. [12], and Xiong, Chen, He, Guan and Chen [2] further introduced resident travel data, such as transit IC card data and existing bus stops to design community shuttle services to provide travelers with more accurate services. However, the transit IC card data only record the process between pick-up and drop-off stops without the activities before going to the stops. Moreover, designing shuttle routes based solely on transit data and bus stops introduces deviations from actual travel patterns, resulting in an inadequate match between services and demands.

Therefore, it is necessary to analyze travel patterns based on resident mobility OD data to better respond to demands. More importantly, these studies lack an assessment of the potential utility of setting up shuttle services, especially the contribution to the surrounding environment. It is well-known that ‘eco-friendly transportation’ is the dominant theme of urban transport development, and environmental assessment can provide important references for transportation policymakers and environmental managers [13].

The intuition is that the development of community shuttle services can reduce private trips and thus directly reduce motor vehicle pollutant emissions. On the other hand, the reduction in private trips can alleviate traffic congestion, which further indirectly reduces unnecessary pollutant emissions from other vehicles due to traffic congestion (e.g., low traffic speed with frequent acceleration and deceleration [14]).

To this end, we propose a complete framework for evaluating the positive impact of community shuttle services, including route design to maximize travel demand, fine-grained quantitative assessment of congestion improvements, and potential analysis of environmental protection. Specifically, the main contributions of this paper are:

(1) We develop a novel method to adaptively generate shuttle stops with maximum service capacity based on crowd movement data, and design shuttle routes with minimum mileage by genetic algorithm;

(2) By identifying trips that can be shifted to shuttle services, we conduct a fine-grained quantitative assessment of the extent to which community shuttle services alleviate traffic congestion and reduce traffic-related air pollution;

(3) Provide guidelines for governments and policymakers to analyze the environmental benefits of setting up eco-friendly transportation and support detailed policymaking regarding transportation environmental issues.

The remainder of this paper is organized as follows: Section 2 summarizes related work from the aspects of community shuttle service design and traffic-related environmental assessment. Section 3 introduces the data, study area, and methods. Section 4 analyses the results. Section 5 discusses this work. Finally, Section 6 concludes this paper.

## 2. Related Works

### 2.1. Traffic-Related Environmental Evaluation

The urban environment is progressively becoming worse due to the significant expansion of vehicle numbers. Recently, more attention has been paid to monitoring and improving traffic air quality. Traditional approaches using air monitoring stations are constrained by station location, coverage range, and construction cost.

Motivated by the availability of human mobility data, researchers have attempted to estimate traffic emissions at larger spatiotemporal scales using floating-car trajectory data, ride-hailing order data, cellular data, etc. For example, Misra et al. [15] estimated urban traffic emissions using vehicle type distribution data and an integrated modeling approach. Sun et al. [16] inferred an entire traffic flow using trajectory data and further estimated vehicular fuel consumption and emissions using the comprehensive modal emissions model (CMEM). Kan, Tang, Kwan and Zhang [3], Kan et al. [17] estimated fuel and emissions at a fine-grained level based on an analysis of vehicle mobile activities, which achieved satisfactory accuracy.

Furthermore, researchers analyzed the influence of different traffic regulations on traffic emissions. Panis et al. [18] and Kumar et al. [19] studied the effect of active speed management on traffic emissions. Ma et al. [20] and Chen and Yuan [21], studied the relationship between traffic signal control strategies and traffic emissions. Mahmod et al. [22] studied the impact of local traffic measures, such as traffic demand control and banning heavy-duty vehicles, on reducing emissions. These studies help traffic operators design environmentally friendly traffic policies and strategies.

In recent years, with the emergence of the concept of a ‘sharing economy’, traditional traffic patterns have changed to some extent. Shared mobility encourages the sharing of transportation resources and an increase in vehicle utilization, thereby reducing vehicles and associated emissions. Overall, emerging shared mobility models can be summarized into mainly three categories [5,23,24,25]:

(1) Vehicle sharing [26,27] remains incapable of providing on-demand and point-to-point ride services, and its environmental impact is uncertain [5];

(2) Ride-sourcing [23,28] refers to transportation services connecting community drivers with passengers via mobile applications. Ridesharing has been expanding rapidly across the world in recent years, with many successful Transport-Network-Companies (TNCs), such as Uber and Lyft in the U.S., DIDI in China, Ola in India, and Grab in Southeast Asia;

(3) Ride-sharing [29,30], allows multiple passengers with similar origins and destinations to share a ride. Studies have proven that ridesharing is not only practical to provide on-demand services, but also effective in reducing Vehicle-Miles-Travelled (VMT), thereby reducing traffic emissions.

However, current studies mainly focus on evaluating the overall benefits of shared mobility, which are good to endorse new mobility paradigms, but clearly insufficient to support detailed policymaking regarding transportation environmental issues [5]. Accordingly, detailed and targeted policies require fine-grained emission estimation at the road-segment level, rather than overall aggregation [26].

On the other hand, all types of shared mobility per se are not sufficient to perfectly achieve these benefits. Any trips that meet each individual’s travel requirement, such as delayed tolerance and time window, can be pooled but may not result in significant reductions in VMT and emissions [5]. Instead, community shuttle services, as flexible, emerging means of transportation, can better relieve traffic congestion and provide cost-effective and eco-friendly transportation for citizens.

### 2.2. Community Shuttle Service Design

Community shuttle service, also called feeder bus service, is a flexible public transit mode used to ferry travelers to or from transportation hubs. Designing public transit is a complicated process that mainly comprises route network design, frequency setting, timetabling, vehicle scheduling, and crew scheduling [31]. Route network design and frequency setting influence the overall operational efficiency of the public transport system [32] and determine passenger choices for public transit [2]. Kuah and Perl [33] formally defined the Feeder Bus Network Design Problem (FBNDP) using a mathematical programming model in which the route and the frequency are optimized with the objective function of minimizing the total costs. Previous studies have focused on the design of shuttle networks, which can be classified as a vehicle routing problem (VRP). Lúcio Martins and Vaz Pato [34], Shrivastav and Dhingra [35], and Kuan, Ong and Ng [8] solved the problem using heuristic algorithms, such as the genetic algorithm, tabu search algorithm, and ant colony algorithm. Ibaraki et al. [36] further considered capacity and time-window constraints and modeled the community shuttle service design as the vehicle routing problem with time windows (VRPTW). Unfortunately, these studies took shuttle stops as pre-given and ignored the location decision of stops, which directly determines the accessibility of shuttle services.

Considering locating shuttle stops, Xiong et al. [37] and Guo, Song, He, Bi and Jin [32] selected candidate stops and generated optimal schemes using an optimization algorithm. However, due to the lack of demand investigation, the shuttle services in these studies are not demand-responsive. With the increasing amount of residents’ mobility data, researchers further introduced them to route service design, aiming to fully meet travelers’ demands. Wang et al. [38] established the potential demand model of roads and generated routes covering most of the regions with traveling demands. Kong, Li, Tang, Tian, Moreira-Matias and Xia [11] predicted travel demands using transit IC card data and planned dynamic routes that can attract more passengers than the existing routes. Xiong, Chen, He, Guan and Chen [2], and Wu et al. [39] further improved the design by considering headway and fuel consumption.

### 2.3. Research Gap

Although the above studies have discussed the issues of the community shuttle service design and the environmental impact evaluation of transportation modes, to the best of our knowledge, few studies have addressed the impact assessment of community shuttle services on traffic and traffic-related air pollution, let alone refined and quantified emission estimates at the road segment level. In addition, the studies on route network design mainly used existing bus stops as candidate shuttle stops and analyzed traffic demands using transit IC card data. These existing bus stops are not set up based on crowd mobility data and may not exactly match the travel demands of residents. Additionally, the IC card cannot record human activities before the arrival at the stops, leading to deviations in demand analysis, which is important for last-mile service [2].

Therefore, (1) how to design stations to meet as many short-distance trips as possible and increase the shifting ratio of eco-friendly transportation; and (2) how to quantitatively assess the potential of community shuttle services to improve road traffic and the surrounding environment are urgent issues to be addressed and are also critical topics for urban sustainable development.

Motivated by this intuition, we designed a route network based on residents’ mobility data and finely evaluate its potential impact on traffic and traffic-related air pollution to support detailed policymaking regarding transportation environmental issues.

## 3. Methods

In this section, we introduce our study area, steps for shuttle route design, and methods for evaluating improvements to traffic and the surrounding environment.

### 3.1. Study Area and Residential Mobility Data

**Figure 1:**
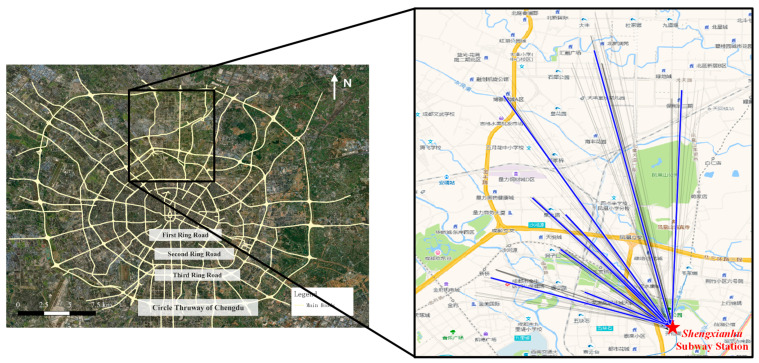
Study area and dominant mobility trends.

The study area (shown in Figure 1) is in the northern area within the 2nd Ring Road and the Circle Thruway of Chengdu, China, which can be represented by longitudes from 104.0406 to 104.1124° E and latitudes from 30.6970 to 30.7713° N in the GCJ-02 Coordinate System. A large number of residential blocks are located in this region; however, the subway network is relatively underdeveloped. According to a previous study [40], numerous residents are traveling by ride-hailing from those residential blocks to only one subway station in the area, namely Shengxianhu Station, forming travel clusters. These massive private trips also release traffic emissions that further pollute surrounding residential areas around roads. Therefore, community shuttle services are needed in these areas to provide convenience and reduce traffic pollutant emissions for residents.

We adopted the ride-hailing order dataset in November 2016 within the study area as the resident OD data (https://sts.didiglobal.com, accessed on 16 November 2022). The data were provided by DiDi, the dominant ride-hailing service provider in China. DiDi covers a number of businesses such as cabs, private cars, and hitchhiking, enabling convenient online cab-hailing services. Therefore, DiDi data can reflect residents’ self-driving or taxi-hailing travel patterns to a certain extent. Moreover, there are 8.5 million DiDi users in Chengdu, accounting for 60% of the resident population. The large number of users enables the data to reflect the spatiotemporal pattern of resident trips accurately and completely [41].

The dataset of ride-hailing trip records includes the order ID, latitude, longitude, and time of origin and destination of each trip. An example of the data records is shown in Table 1, where “Time-d” and “Time-a” represent departure time and arrival time, respectively (in UTC seconds); “Longitude-d” and “Latitude-d” are the geographic locations of the departure point; and “Longitude-a” and “Latitude-a” are the geographic locations of the arrival point (in the GCJ-02 Coordinate System).

**Figure 2:**
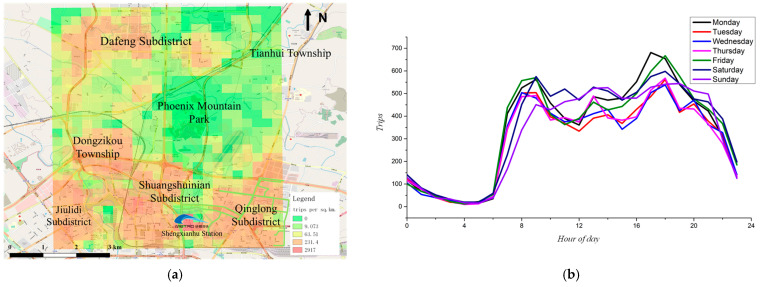
Spatiotemporal distribution of the dataset in the study area. (**a**) Spatial distribution of O/D points; (**b**) number of trips per hour.

As shown in Figure 2, massive trips are concentrated in large residential areas, especially near and within the 3rd Ring Road; the curve of trips by hour shows that there are more than 300 trips per hour from 7 a.m. to 10 p.m. every day with a similar pattern and stable ridership on both weekdays and weekends. In addition, the average traveling time of trips in the study area is 12.73 min, and 82.73% of the traveling time is less than 20 min. This shows that trips in the study area are dominated by short trips. In summary, the study area is suitable for setting up community shuttle services from 7 a.m. to 10 p.m.

### 3.2. Designing the Community Shuttle Route Based on Resident OD Data

In what follows, we provide the definition and design criteria of community shuttle routes.

**Definition 1** **(Community Shuttle Service).** *Community Shuttle Service is a flexible public transit mode to transfer passengers between the transportation hub and communities. It consists of a station, stops, and routes. The station is the principal place of departure or arrival and is always the nearest transportation hub to the residences, such as a subway station or bus terminal. Stops are the sites along roads to pick up or drop off passengers. Routes are the paths that shuttles travel in a circle. On each route, the shuttle departs from the station, drives through several stops, and returns to the station.*

**Definition 2** **(Shuttle Service Capability).** *Shuttle service capability refers to the ability to meet residents’ travel demands. Please note that, in this paper, capacity denotes how many passengers a shuttle bus can accommodate, and is a static indicator; while service capability focuses on the extent to which the shuttle can meet the travel needs of residents. Thus, the desirable shuttle stops and routes should be designed to meet as many residents’ travel needs as possible, thereby increasing the shifting ratio of low-carbon transportation.*

Stop location and route design are the two main decisions in planning a community shuttle service. These two decisions are interrelated and interact, and are strongly related to the user cost and operating cost. The optimal stop location and the route can help reduce the walking distance of passengers and the route length. Thus, the aim of designing community shuttle services is to cover the demands of the majority of passengers in the area with the least construction and operation costs, namely, with the shortest total route distance and the fewest stops. In what follows, we optimize the specific solution for community shuttle service in two aspects: generating shuttle stops and devising shuttle routes.

#### 3.2.1. Generating Shuttle Stops with Maximized Service Capability

There are some constraints that need to be followed in terms of the generation of shuttle stops.

**Optimization Objective 1: Maximize service capability.** Each stop serves travelers whose walking distances to the stop are lower than a maximum tolerable value; thus, the distribution of stops needs to cover as many travelers as possible.

**Optimization Objective 2: Minimize the number of stops.** Community shuttle services should be designed to minimize the number of stops while ensuring maximum service capacity to reduce operating costs.

**Constraint 1-1: Stops location constraint.** To facilitate shuttle bus parking and pass-through freely, stops must be located along roads.

**Constraint 1-2: Station distance constraint.** A minimum distance constraint should exist between adjacent stops to reduce the number of stops and shorten the time decay at shuttle stops.

**Figure 3:**
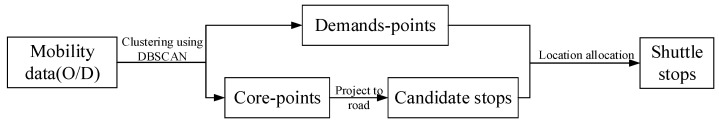
Flowchart of generating shuttle bus stops.

To design a community shuttle service, we first generated stops based on mobility data with the target of maximizing the service capability. Considering the service coverage constraint and stop construction cost, it is desirable to allocate stops reasonably to meet the traveling demands using a minimum number of stops. To achieve this, we developed a method for generating stops based on spatial analysis approaches. The flowchart of the method is shown in Figure 3.

***Step 1: Identify locations with demands for shuttle services***

Bus stops are usually set at locations with dense passenger O/D points to maximize service capacity. Therefore, we first distinguished areas with dense passengers using the DBSCAN method [42]. As a commonly used spatial clustering method, DBSCAN can find clusters and identify noise by the neighboring object numbers within a given distance. In the result, the noise points, namely locations with fewer passengers, are filtered out and the clustered points are considered locations with demands of shuttle services.

***Step 2: Generate candidate shuttle stops from core-points***

According to **objective 1** and **constraint 1-1**, shuttle stops should be within the O/D hotspot areas and along the roads. Thus, in this study, we project the clustered O/D points onto the nearest road as the candidate stops. Furthermore, the distance constraint (**constraint 1-2**) should be satisfied between shuttle stops, i.e., overlapping or close points can be eliminated or merged to reduce operating costs.

***Step 3*** ***: Generate the optimal stops scheme by location-allocation***

The optimal stops scheme from candidate stops, which can cover all the demands with the minimum amount (**objective 2**) and a fixed service radius, is further generated using the location-allocation method. For this step, the location-allocation tool, provided by ArcMap software, is used in “Minimize Facilities” mode. The tool builds the matrix of network distances between the candidate stops and demand points and then solves the problem using heuristic algorithms to find an optimal scheme within a nearly infinite number of solutions. The detail of the tool can be referred to Esri [43].

#### 3.2.2. Generating Shuttle Routes by Genetic Algorithm

Overall, based on the obtained shuttle stops, the design of shuttle routes needs to meet the following conditions.

**Optimization Objective: Minimize mileage.** Ensuring service capacity and operation cost, the principle of generating shuttle routes is to connect all stops to the station without any transfer or connection while minimizing the mileage traveled [33,44], i.e.,

(1) $$\min\sum_{k}mileage_{k},\forall k\in K$$

where *mileage_k_* denotes the miles of the *k*th route, and *K* is the total number of shuttle routes.

**Constraint 2-1: Subway connection constraint**. With the function of shuttle service, each route needs to connect to at least one subway station [2,32], i.e.,

(2) $$\sum_{stop_{i}\in route_{k}}I(stop_{i}=sub)\geq 1,\forall k\in K$$

where *route_k_* denotes the set of all stops of the *k*th route, *stop_i_* represents the *i*th shuttle stop, and *sub* denotes the subway station. The *I* function is an indicator function that takes value 1 when the condition meets, otherwise it takes value 0.

**Constraint 2-2: Route distance constraint.** The driving distance of each route has to be within a reasonable range, mainly determined by the network scale and vehicle energy [2], i.e.,

(3) $$\delta_{\min}\leq NetLen_{k}\leq \delta_{\max},\forall k\in K$$

where *NetLen_k_* denotes the total length of route *k* in the road network; and *δ_min_*, *δ_max_* are the minimum and maximum thresholds of route distance, respectively.

**Constraint 2-3: Number of stops on each route.** Buses stop for a while at each stop to pick up and drop off passengers. Therefore, the number of stops on each route needs to be within a reasonable range to ensure travel efficiency, i.e.,

(4) $$\sigma_{\min}\leq \left|route_{k}\right|\leq \sigma_{\max},\forall k\in K$$

where |*route_k_*| denotes the cardinality of stop set *k*, i.e., the total number of stops of route *k*; *σ_min_* and *σ_max_* are the thresholds for the number of shuttle route stops.

To this end, we chose the only subway station, Shengxianhu Station, as the shuttle station, and utilize the yielded shuttle stops to generate the route with the minimum mileage. We employed the genetic algorithm to solve this optimization problem [38,45]. The genetic algorithm, which has been widely used in public transit network design, contains multiple crossover and mutation operators to guide the evolving process of generating feasible solutions, and the convergent optimal solution would be the final route. The specific steps for generating shuttle routes using the genetic algorithm are provided in Algorithm 1, and more details can be referred to Mohammed, Abd Ghani, Hamed, Mostafa, Ahmad and Ibrahim [9], Kong, Li, Tang, Tian, Moreira-Matias and Xia [11], Wang, Wen, Yi, Zhu and Sun [38].
**Algorithm 1.** Designing Shuttle Bus Routes with Genetic Algorithm**Input:** generated shuttle stops, distance matrix, iteration epochs *maxiter*, crossover probability *pc*, mutation probability *pm*, population size *pop_size*, population selection probability *p_select*.
1Initialize populations to the default, i.e., generate random sequences for all shuttle stops.2Based on **Constraints 2-1 to 2-3**, split and generate the initial sets of shuttle routes.3Calculate the length and fitness of the initial route set, fitness = 1/sum of routes length.4Randomly select an initial set of shuttle routes as the best chromosome.5Loop for *maxiter* iterations, in each iteration:5.1Population selection, retaining populations (shuttle routes sets) with high adaptation, i.e., route sets with short mileage.5.2If crossover conditions are satisfied (*pc* and *pop_size*), do crossover between chromosomes of two populations: 5.2.1Randomly select two crossover stops in the range “2” to “No of stops”.*//Note: The first stop is fixed, namely Shengxianhu Station.*5.2.2Perform chromosome crossover and ensure that there are no duplicate stops in the same set of shuttle routes.5.3If mutation conditions are satisfied (*pm* and *pop_size*), do mutation for each chromosome.
5.3.1Choose two random numbers [a], [b] in the range “2” to “No of stops”.5.3.2Swap the values of new gene [a] and new gene [b].5.4Based on **Constraints 2-1 to 2-3**, split and generate a new set of shuttle routes.5.5If the fitness value of the new route set is greater than that of the best chromosome, then set best chromosome = new chromosome.6End of Loop.
**Output:** The best set of shuttle bus routes.

### 3.3. Analyzing the Shifting Potential and Environmental Impacts

#### 3.3.1. Condition of Shifting Traveling Mode to Community Shuttle Service

In general, the shift from traveling by self-driving or taxi-hailing to public transit depends mainly upon walking distance, traveling time, and fare [6,32]. The fare varies according to different policies or concessions, but the walking distance and traveling time depend on the service capability. Therefore, to evaluate the potential ridership of the community shuttle service, we ignore the influence of other subjective factors and determine whether riders can shift modes to the shuttle service based on walking distance and traveling time ratio, calculated as Equations (5)–(7).

(5) $$dis(O_{i},stop_{j})\leq twd$$

(6) $$dis(D_{i},stop_{k})\leq twd$$

(7) $$t_{i}^{shuttle}\leq t_{i}^{private}\cdot ratio$$

where *O_i_* and *D_i_* represent the O/D positions of the *trip_i_*; *stop_j_* and *stop_k_* represent the nearest shuttle stops of *O_i_* and *D_i_*, respectively; *twd* represents the tolerable walking distance to the shuttle stops; $t_{i}^{shuttle}$ and $t_{i}^{\;private}$ represent the total traveling time of the *trip_i_* by shuttle service and private mode (such as self-driving and taxi-hailing), respectively; and *ratio* represents the tolerable longest time of $t_{i}^{shuttle}$ over *i*.

The total traveling time for the shuttle service consists of four components: vehicle travel time $t_{i}^{travel}$, walking time $t_{i}^{walk}$ between the stop and the actual O/D location, time decay $t_{i}^{stops}$ for passing stops, and time waiting $t_{i}^{wait}$ for the shuttle, which can be formed and calculated as (8)–(12):

(8) $$t_{i}^{shuttle}=t_{i}^{travel}+t_{i}^{walk}+t_{i}^{stops}+t_{i}^{wait}$$

(9) $$t_{i}^{travel}=d_{i}/v_{bus}$$

(10) $$t_{i}^{walk}=(dis(O_{i},stop_{j})+dis(D_{i},stop_{k}))/v_{walk}$$

(11) $$t_{i}^{stops}=N_{i}\cdot t_{d}$$

(12) $$t_{i}^{wait}=0.5\cdot t_{h}$$

where *v_bus_* and *v_walk_* represent the average speed of the shuttle bus and walking, respectively; *d_i_,* and *N_i_* represent the distance traveled and the number of stops passed by trip *i*, respectively; *t_d_* and *t_h_* represent the time decay at each stop and the headway of the shuttle bus.

Referring to previous studies and design standards [2,7,46,47], we set the parameters as follows: *v_bus_* = 30 km/h, *v_walk_* = 5 km/h, *t_d_* = 60 s, *t_h_* = 5 min, *twd* = 500 m. Due to the lack of references on the tolerable time ratio for shifting from private mode to shuttle service, we refer to the subway travel parameter and set it to 3 [48].

#### 3.3.2. Estimating Traffic Distribution on the Road Network

**Figure 4:**
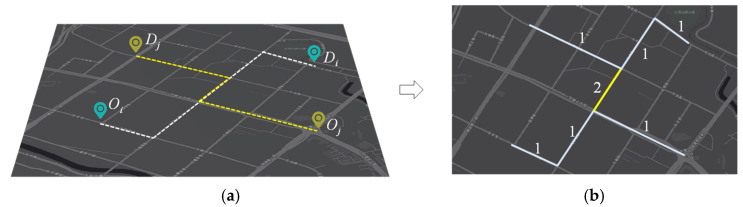
Estimation of traffic distribution on the road network. (**a**) Restore the trip path by the shortest path; (**b**) calculate traffic volume distribution, where the numbers indicate traffic volumes.

To evaluate the potential effect on road segments, we estimate and compare the traffic volume before and after setting up the community shuttle service. Specifically, due to the lack of actual travel paths in the dataset, we assume that each ride-hailing trip follows the shortest path within its O/D locations. Then, as shown in Figure 4a, the shortest path is generated using the Dijkstra algorithm [49] to distribute traffic flows and restore the passing road segments. Finally, as shown in Figure 4b, the traffic volume in each segment is the count of trips passing on it. The change in traffic is defined as the difference between travel volume from the total number of trips and trips potentially to be shifted to community shuttle services.

#### 3.3.3. Estimating the Impact of Traffic Emissions on the Surrounding Environment

Traffic emissions can be estimated mainly at both macro and micro levels. Macroscopic models use aggregate methods to analyze the total emissions in spatial units based on average speeds and fixed emission factors. In contrast, microscopic models generally use agent-level models to estimate traffic emissions per vehicle on each road segment or intersection with dynamic emission factors calculated using speed fluctuations [5].

The COPERT III model [50] is a typical microscopic model designed to estimate the emissions of vehicles that comply with European emission standards. Recently, the COPERT III model was calibrated and validated by Xie et al. [51], Shang et al. [52] based on actual categories, driving cycle, and fuel characteristics of Chinese vehicles, providing valid equations for calculating dynamic emission factors, which has been implemented in many studies [3,5,17].

In the COPERT III model, traffic emissions consist of three parts: hot, cold start, and evaporative emissions. Hot emissions occur when the engine is at its normal regime, which is the general condition for a running vehicle and thus is our major concern. Cold start emissions denote emissions from transient engine operation, and evaporative emissions come from refueling and temperature changes. Following Yan, Luo, Zhu, Santi, Wang, Wang, Zhang and Ratti [5], Shang, Zheng, Tong, Chang and Yu [52], the latter two components are omitted in our estimation due to their less significance in terms of overall emissions.

The hot emission factor (*EF*) for a specific pollutant *k*, i.e., the amount of pollutant *k* emitted by a single vehicle per kilometer, is a function of travel speed *v* (km/h), as in Equation (13).

(13) $$EF_{k}(v)=(a_{k}+c_{k}v+e_{k}v^{2})/(1+b_{k}v+d_{k}v^{2}),$$

where *EF_k_* (*v*) represents the hot emission factor of pollutant type *k* at an average speed of *v*; and *a_k_, b_k_, c_k_, d_k_,* and *e_k_* are factors of pollutant *k*.

According to local ride-hailing management policies, the ride-hailing vehicle is allowed under engine displacement of 1.6 L (naturally aspirated) or 1.4 T (turbocharging); therefore, the parameter in the COPERT III model is shown in Table 2. As for other pollutants, such as CO_2_ and PM_2.5_, the emission factors are proportional to fuel consumption (FC). For example, the conversion factor for CO_2_ is 3.18 and its emission factor can be expressed as:

(14) $$EF_{CO_{2}}=3.18\times EF_{FC}.$$

Accordingly, the cumulative emission of pollutant type *k* on road segment *i* is:

(15) $$E_{i,k}=EF_{k}(v)\cdot N_{i}\cdot Len_{i},$$

where *N_i_* and *Len_i_* represent the number of vehicles passing through road *i* and the length of road *i*, respectively.

Further, traffic emissions spread with air motion and pollute the road and surrounding environment, and their impact decreases with increasing distance from the road centerline [53,54]. Therefore, for precise estimation of the potential impact of the community shuttle service on the surrounding environment, this study adopts the line feature kernel density estimation method to model the concentration of pollutants around the road network.

**Figure 5:**
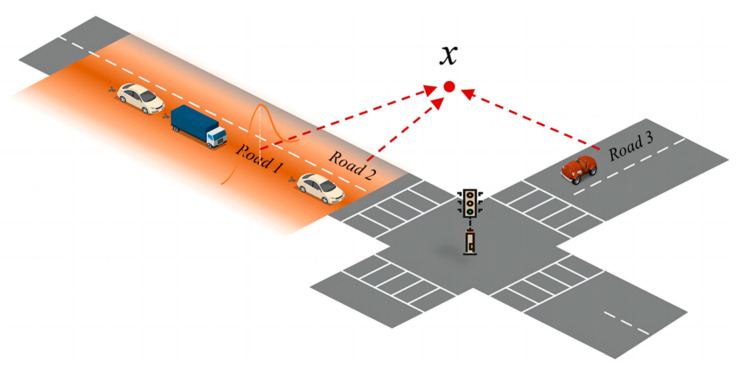
Effect of traffic emission dispersion on concentration at location *x*.

Specifically, Figure 5 demonstrates the impact of traffic emissions on the surrounding environment. We assume that the pollutant concentration at location *x* is influenced by Roads 1, 2, and 3. In this paper, we utilize *dis*(*x*, *r_i_*) to denote the distance between location *x* and road centerline *r_i_*; and employ kernel function *K*(·) to represent the functional relationship of decreasing influence with increasing distance. To this end, the kernel density of location *x* can be calculated as Equation (16).

(16) $$f(x)=\frac{1}{nh}\sum_{i=1}^{n}K\left(\frac{dis(x,r_{i})}{h}\right)\cdot w_{i},$$

where *n* denotes the number of bi-directional road segments that have impacts on location *x*. In Figure 5, *n* = 3 for location *x*. *h* denotes the bandwidth; and *w_i_* is the weight of road *r_i_*, which represents the pollutant emission on road segment *i* in this paper.

According to Gordon, Staebler, Liggio, Li, Wentzell, Lu, Lee and Brook [54], Pratt et al. [55], the traffic air pollutant intensity attenuates outward from the centerline of roadways and reaches the background value of approximately 300 m; thus, the bandwidth is set as 300 m in this paper.

## 4. Results

### 4.1. Community Shuttle Service Design

#### 4.1.1. Generation of Shuttle Stops

**Figure 6:**
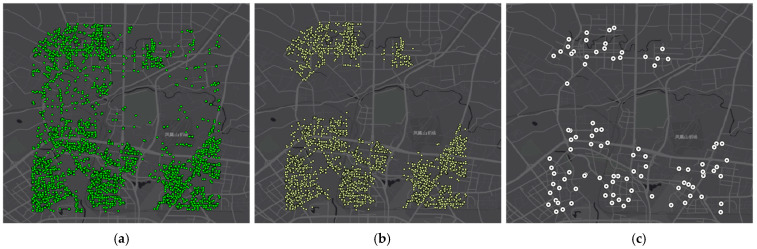
Shuttle stops generation. (**a**) All O/D locations; (**b**) clustered locations; (**c**) generated shuttle stops.

Experiments are performed using travel data in November 2016. Taking November 1 as an example, Figure 6 shows the process of generating shuttle stops. The residential mobility data include 9310 O/D points (Figure 6a). A total of 97 shuttle stops are generated, covering 7270 O/D points within 500 m walking distance, meeting 78.09% of the total traveling demand (Figure 6b). Figure 6c shows the generated shuttle stops.

**Figure 7:**
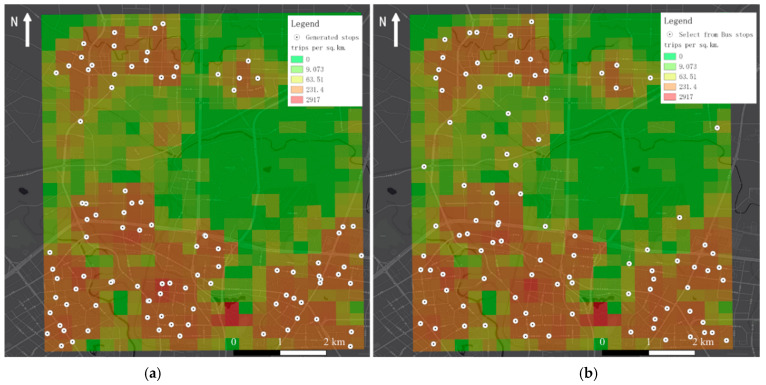
Generated shuttle stops with our method and comparison. (**a**) Generated shuttle stops with our method; (**b**) selected stops from existing bus stops.

As mentioned above, DIDI data can reflect crowd movement trends to some extent. In what follows, we further compare the generated stops with existing bus stops to illustrate the advantages of the adaptive stop generation method based on the actual travel data, and the results are shown in Figure 7.

Comparing the two scenarios, almost all stops generated by our method are located in travel hotspots, while some existing bus stops are located at locations with fewer trips. Furthermore, we quantitatively evaluate the average number, standard deviation (STD), and the total percentage of OD points covered by each stop in both scenarios, and the results are shown in Table 3.

According to the results, the higher average and cover percentage of the proposed scheme indicates that our method can cover more travel demands, thus minimizing private trips; while the lower standard deviation indicates that our results have a more balanced service capability and guarantee transportation equity.

#### 4.1.2. Generation of Shuttle Routes

**Figure 8:**
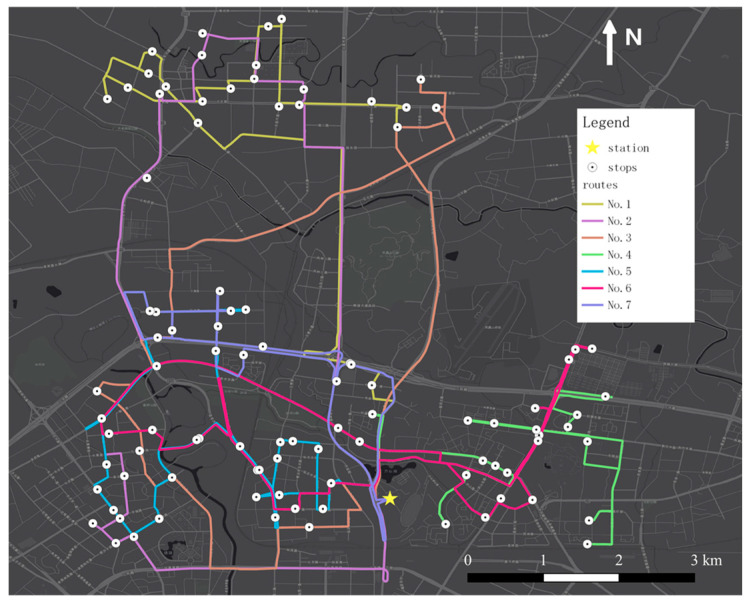
Generated shuttle service network.

Based on the generated shuttle stops, we utilized the genetic algorithm to design shuttle routes. During the generation process, the maximum distance of each route and the maximum stops on each route are set as 30 km and 20, respectively. The generated shuttle network and the details of each route, including the total number of stops, route length, and main service area, are provided in Figure 8 and Table 4.

**Figure 9:**
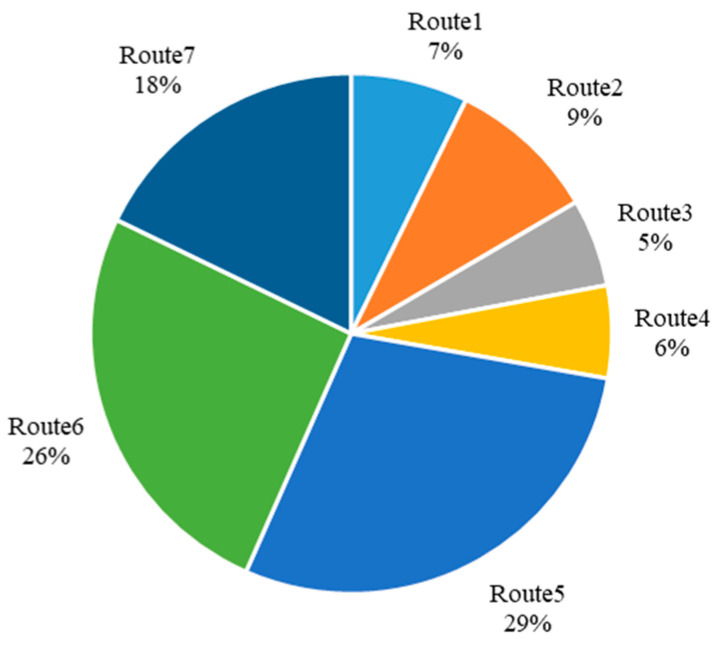
Shuttle service ridership distribution.

We further analyzed the potential ridership of each route. As shown in Figure 9, three routes, Route 5, Route 6, and Route 7 have significantly higher ridership than others, accounting for the majority of the total. This is because the service areas of the three lines are close to or within the 3rd Ring Road. These areas have denser residences, business areas, and O/D points and are closer to the subway station. In contrast, the service areas of Route 1, Route 2, Route 3, and Route 4 are mainly in the northern and western parts of the study area, which is relatively far from the station, and the residential areas are relatively sparse. Therefore, it may take too long to travel to the subway station by community shuttle services in these areas, which reduces the possibility of shifting from private modes.

**Figure 10:**
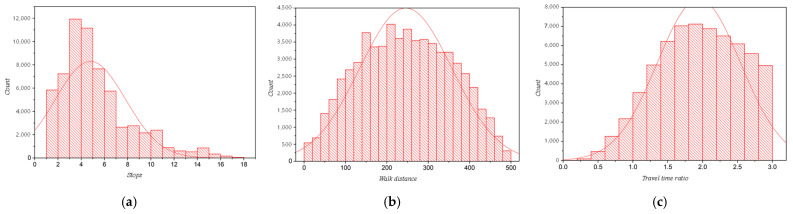
Response of shuttle routes to residents’ demands. (**a**) The number of stops taken; (**b**) walking distance; (**c**) ratio of travel time.

To better represent the possibility of shifting to community shuttle services, Figure 10 presents the number of shuttle stops residents need to take (Figure 10a), the distance they need to walk (Figure 10b), and the ratio of travel time (Figure 10c). In general, it takes residents an average of 4.67 stops to reach their destinations, a walking distance of 245.9 m, and 0.95 times longer than ride-sharing services, which we deem to be tolerable in most cases.

### 4.2. The Potential Reduction in Traffic Volume

**Figure 11:**
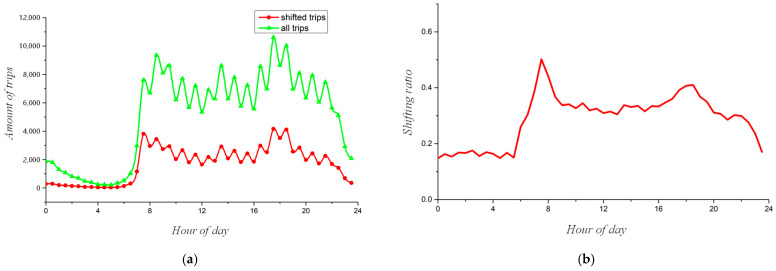
Potential amount and ratio of shifting from ride-hailing to shuttle service. (**a**) The potential number of trips; (**b**) shifting ratio.

Evaluated by the resident OD data, 30.36% of the trips by private mode can be potentially shifted to community shuttle services. Specifically, the shifting ratio and number of these trips are aggregated at half an hour, and the results are shown in Figure 11. It shows that the trends of potential shuttle trips and total trips are similar. There are both more shuttle trips and more total trips in the daytime than at night, with morning and evening peaks. Thus, the operators of shuttle services can adjust the service capability according to the trends, for example, by increasing the frequency of departure during peak hours or adopting higher-capacity vehicles. The shifting ratio reaches 50.2% during the morning peak hours and 41.01% during the evening peak hours and approximately 20% during the night; thus, shuttle service plays a more important role during the daytime and is especially convenient for commuters. This phenomenon is caused by the regularity of traveling purposes of the residents. During the morning and evening peak hours, most residents travel between their residential areas and the subway station, while at night the O/D locations are relatively heterogeneous and random, such as bars and malls.

**Figure 12:**
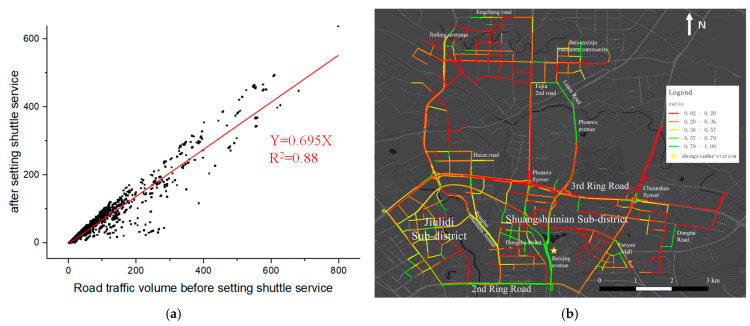
The potential reduction in traffic volume. (**a**) Relationship of traffic volume; (**b**) reduced ratio distribution.

Furthermore, we analyzed the change in traffic volume on each road segment and its spatial distribution at a more fine-grained scale. The relationship between traffic volumes in each segment before and after setting shuttle service is shown in Figure 12.

First, in Figure 12a, the traffic volumes before and after setting up shuttle services are positively correlated at the 0.05 significance level with a slope of 0.695, indicating that the traffic pressure on all road segments has been alleviated by 30.5% on average. In Figure 12b, the Jenks natural breaks method [56] is used to categorize the ratio values into gradually varied colors.

As can be seen, the traffic volumes within two sub-districts located between the 2nd Ring Road and 3rd Ring Road, namely Jiulidi and Shuangshuinian Subdistricts, are significantly reduced, with the ratio of over 36%; the traffic volumes on some traffic arteries heading to the Shengxianhu station, such as 2nd Ring Road, Beixing Avenue, snf Dongsha Street, are reduced significantly by over 57% of original traffic volume; the traffic pressure of some transportation hubs and passageways, such as Jinfeng overpass, Chuanshan flyover, Phoenix flyover, Phonenix avenue, and Rongbei business avenue, is also significantly relieved. The reduced traffic volume may improve traffic efficiency and alleviate traffic congestion, thus reducing unnecessary pollutant emissions.

### 4.3. The Potential Contribution to the Surrounding Environment

The overall reduction in emissions is shown in Table 5. Since the travel speed is regarded as a constant in the emission estimation, the reduced ratios are the same. According to the results, the community shuttle service is able to reduce the daily pollutant emissions in the area by about 28%; specifically, about 1.66 tons of CO_2_ emissions can be reduced. It is obvious that such eco-friendly transportation can decrease greenhouse gas (GHG) emissions and promote sustainable urban development.

**Figure 13:**
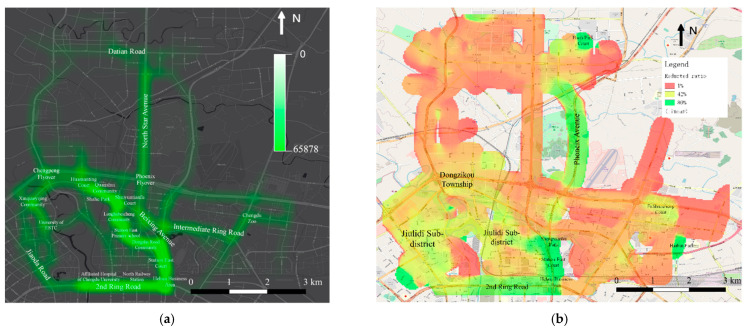
Spatial distribution of reduced emissions by setting up the shuttle service. (**a**) Spatial kernel density of reduced emission; (**b**) spatial distribution of reduced emission ratio.

Furthermore, taking CO as an example, we analyzed the reductions of pollutant emissions at the road-segment level (Figure 13a), and evaluate the impacts on the surrounding environment using the line feature kernel density estimation method (Figure 13b). Although the reductions of different pollutants vary, their spatial distribution and reduction ratio are approximately the same.

In Figure 13a, the traffic emissions in the areas along the main roads, such as North Star Avenue, Intermediate Ring Road, 2nd Ring Road, Datian Road, and Beixing Avenue, have been significantly reduced. More obviously, there is a strip with a high reduction from the Huamanting Court to the Affiliated Hospital of Chengdu University, which will benefit the residents of Quanshui Community, Longhubeicheng Community, Dongsha Road Community, Station East Court, and public facilities, such as Shahe Park, Station East Primary School, North Railway Station, and Affiliated Hospital of Chengdu University. In Figure 13b, the traffic pollution around Phoenix Avenue, 2nd Ring Road, is largely eliminated with a percentage of approximately 80%; the traffic pollution in several large residential communities within the southwest side of the study area is also effectively controlled, with a reduced percentage of approximately 42%.

In summary, the community shuttle service, as an eco-friendly transportation mode, has the potential to reduce traffic pollution and GHG emissions by nearly half within some residential areas.

## 5. Discussion

From the case study in Chengdu, we find that 30.36% of private trips have the potential to be replaced by community shuttle services. The percentage even reaches 50.2% during peak hours.

It should be noted that this study only utilizes ride-hailing order data as resident mobility data, which, despite its representativeness, is not comprehensive and complete enough. Admittedly, obtaining comprehensive resident mobility data is not a simple task. Thus, in this paper the ratios are more instructive than the quantitative values. It is worth mentioning that Yu et al. [57] quantitatively evaluated the potential of energy savings and emission reduction in customized buses through mobile phone GPS data.

In terms of shifting potential assessment, we set some properties, such as vehicle capacity, headway, and vehicle speed, as fixed values by referring to the existing literature, which ignores the influence of various values on the potential of the shuttle service. Admittedly, single-parameter settings are often unreliable. Walk accessibility and tolerance can be influenced by many factors, such as age, comfort, ease of walking, and weather conditions. It is non-transferable from one geographic territory to another. Recently, Xiong, Chen, He, Guan and Chen [2] and Kong, Li, Tang, Tian, Moreira-Matias and Xia [11] improved the operating efficiency of shuttle services by dynamically setting headways, capacities, and even routes synchronously. These strategies can further reduce the waiting time and traveling time and then increase the potential of the shuttle service to a certain extent.

For low-carbon transport advocacy, we only evaluate the potential of the shift to shuttle services based on objective factors, ignoring the influence of subjective factors, such as travel purpose, cost acceptance, and traveler attributes [48]. For example, travelers may prefer to take cars to hospitals regardless of cost. It is an open question how to evaluate the likelihood of travelers shifting to public transit from private modes.

Regarding environmental protection, this paper employs the line kernel density method to evaluate the impact of reduced traffic emissions on the surrounding environment. Please note that the spatial distribution of reduced traffic emissions shown in Figure 13 does not consider the influence of external meteorological conditions. We focus more on the quantitative assessment of the improvement of surrounding environment by low-carbon travel modes. In reality, the spatial distribution of pollutants is affected by not only anthropogenic sources but also other meteorological and biological factors, such as wind advection, temperature, and seasonal biotic fluxes. The reduction in air pollutants may be not only due to the community service shuttle route designed in this study, but also merely due to changes and differences in wind speed and direction or temperature hot spots.

Overall, crowds’ green travel intentions and carbon reduction potential assessments of eco-friendly transportation remain challenging issues. In future studies, we will improve the accuracy and reliability of the potential analysis of shifting to shuttle services by introducing more extensive mobility datasets and considering more factors related to vehicles and traveling purposes.

## 6. Conclusions

Community shuttle services, as flexible public transit modes, have the potential to improve the accessibility of public transportation in newly built areas and improve the road and surrounding environment polluted by vehicles. Existing studies are focused on the methods of service design, and neglect to assess its impact on traffic and traffic-related air pollution.

In this study, we propose a complete framework for evaluating the positive impacts of community shuttle services, including route design to maximize travel demand, fine-grained quantitative assessment of congestion alleviation, and potential analysis of environmental protection. Specifically, we develop a novel method to adaptively generate shuttle stops with maximum service capacity by mining the spatial pattern of resident ride-hailing O/D positions, and design shuttle routes with minimum mileage by genetic algorithm. Then, considering the walking distance and travel time, we identify trips that can be shifted to community shuttle services and their potential changes in traffic flow. In terms of environmental evaluation, we utilize the COPERT III model and the spatial kernel density estimation method to further analyze the reduction in traffic emissions by eco-friendly transportation modes to support detailed policymaking regarding transportation environmental issues.

A case study in Northern Chengdu is carried out, and the results indicate that (1) the generated shuttle stops can better satisfy the travel demands of crowds than existing bus stops, (2) shuttle services can replace 30.36% of private trips and provide convenience for 50.2% of commuters, and (3) shuttle services can reduce traffic emissions by 28.01% overall and approximately 42% in the surrounding residential areas, thus benefiting the health of residents.

Thorough experiments indicate that such eco-friendly transportation can directly reduce GHG emissions from motor vehicles by shifting mobility patterns. Moreover, reducing private trips can alleviate traffic congestion, which further indirectly reduces unnecessary pollutant emissions from other vehicles due to traffic congestion.

To summarize, this paper provides references and guidelines for managers to design and evaluate the environmental benefits of community shuttle services. Nevertheless, the study does not consider the influence of certain factors, such as different vehicle types, headways, and traveling purposes. Further research is needed to make the results more accurate by introducing more mobility datasets and considering the influence of more factors.

## Figures and Tables

**Table 1:** Description of the ride-hailing dataset used in this study.

Order ID	Time-d	Time-a	Longitude-d	Latitude-d	Longitude-a	Latitude-a
eb9dd4095d9850e6287cefd8	1477964797	1477966507	104.0946	30.70397	104.0893	30.65085
387a742fa5a3fbe4a1f215ac5	1477985585	1477987675	104.0765	30.76743	104.0637	30.58951
9cf55f8e6e02a1e0f792df06e	1478004952	1478006217	104.0197	30.68901	104.1053	30.66395
5feeae0307e15203484b9ffce	1477989840	1477991065	104.0361	30.62269	104.0439	30.68232

**Table 2:** Parameters of emission factors for different pollutants in COPERT III model.

Pollutant	*a*	*b*	*c*	*d*	*e*
CO	71.7	35.4	11.4	−0.248	0
NO_X_	9.29 × 10^−2^	−1.22 × 10^−2^	−1.49 × 10^−3^	3.97 × 10^−5^	6.53 × 10^−6^
Hydrocarbon	5.57 × 10^−2^	3.65 × 10^−2^	−1.1 × 10^−3^	−1.88 × 10^−4^	1.25 × 10^−5^
Fuel Consumption (FC)	217	9.6 × 10^−2^	0.253	−4.21 × 10^−4^	9.65 × 10^−3^

**Table 3:** Comparing the service capability of the generated shuttle stops with the existing bus stops.

	Average	STD	Cover Percentage
Generated shuttle stops (Our method)	**68.12**	**57.43**	**78.09%**
Selected from Bus stops	45.97	64.06	47.89%

**Table 4:** Generated shuttle routes.

ID	Number of Stops	Route Length (km)	Main Service Regions
1	15	29.83	Dafeng Subdistrict
2	11	26.63	Dafeng, Jiulidi Subdistrict
3	7	27.96	Tianhui Township, Shuangshuinian Subdistrict
4	16	28.95	Qinglong Subdistrict
5	19	27.32	Shuangshuinian, Jiulidi Subdistrict
6	19	27.85	Shuangshuinian, Bailianchi Subdistrict
7	10	18.99	Dongzikou Township

**Table 5:** Emissions reduced by shuttle service in one day.

Item	Total Amount	Reduced Amount	Reduced Ratio
Cumulative Distance	27,938 km	7825 km	28.01%
CO	13.762 kg	3.854 kg	
NOx	2.255 kg	0.632 kg	
CO_2_	5919.51 kg	1657.95 kg	
Hydrocarbon	0.49 kg	0.138 kg	

## Data Availability

Data may be made available upon reasonable request.
